# Obesity‐induced MBD2_v2 expression promotes tumor‐initiating triple‐negative breast cancer stem cells

**DOI:** 10.1002/1878-0261.12444

**Published:** 2019-03-01

**Authors:** Emily A. Teslow, Cristina Mitrea, Bin Bao, Ramzi M. Mohammad, Lisa A. Polin, Greg Dyson, Kristen S. Purrington, Aliccia Bollig‐Fischer

**Affiliations:** ^1^ Department of Oncology Barbara Ann Karmanos Cancer Institute Wayne State University School of Medicine Detroit MI USA; ^2^ Department of Computer Science Wayne State University Detroit MI USA

**Keywords:** cancer stem cell‐like cells, MBD2, MBD2_v2, obesity, SRSF2, triple‐negative breast cancer

## Abstract

Obesity is a risk factor for triple‐negative breast cancer (TNBC) incidence and poor outcomes, but the underlying molecular biology remains unknown. We previously identified in TNBC cell cultures that expression of epigenetic reader methyl‐CpG‐binding domain protein 2 (MBD2), specifically the alternative mRNA splicing variant MBD variant 2 (MBD2_v2), is dependent on reactive oxygen species (ROS) and is crucial for maintenance and expansion of cancer stem cell‐like cells (CSCs). Because obesity is coupled with inflammation and ROS, we hypothesized that obesity can fuel an increase in MBD2_v2 expression to promote the tumor‐initiating CSC phenotype in TNBC cells *in vivo*. Analysis of TNBC patient datasets revealed associations between high tumor MBD2_v2 expression and high relapse rates and high body mass index (BMI). Stable gene knockdown/overexpression methods were applied to TNBC cell lines to elucidate that MBD2_v2 expression is governed by ROS‐dependent expression of serine‐ and arginine‐rich splicing factor 2 (SRSF2). We employed a diet‐induced obesity (DIO) mouse model that mimics human obesity to investigate whether obesity causes increased MBD2_v2 expression and increased tumor initiation capacity in inoculated TNBC cell lines. MBD2_v2 and SRSF2 levels were increased in TNBC cell line‐derived tumors that formed more frequently in DIO mice relative to tumors in lean control mice. Stable MBD2_v2 overexpression increased the CSC fraction in culture and increased TNBC cell line tumor initiation capacity *in vivo*. SRSF2 knockdown resulted in decreased MBD2_v2 expression, decreased CSCs in TNBC cell cultures, and hindered tumor formation *in vivo*. This report describes evidence to support the conclusion that MBD2_v2 expression is induced by obesity and drives TNBC cell tumorigenicity, and thus provides molecular insights into support of the epidemiological evidence that obesity is a risk factor for TNBC. The majority of TNBC patients are obese and rising obesity rates threaten to further increase the burden of obesity‐linked cancers, which reinforces the relevance of this report.

**Abbreviations**
BMI: body mass index
CSCs: cancer stem cell‐like cells
DIO: diet‐induced obesity
ERα: estrogen receptor alpha
hPSCs: human pluripotent stem cells
IFNγ: interferon‐gamma
MBD2_v2: methyl‐CpG‐binding domain protein 2 variant 2
ROS: reactive oxygen species
SRSF2: serine‐ and arginine‐rich splicing factor 2
TNBC: triple‐negative breast cancer

## 1 Introduction

In the last 25 years, the prevalence of obesity has doubled in 70 countries, including the United States, and nearly one‐third of adults worldwide are now overweight or obese (Afshin *et al*., 2017). The rising obesity pandemic is decidedly concerning because obesity is a known risk factor for an array of chronic, debilitating, or life‐threatening diseases (WHO, 2000), such as rheumatoid arthritis, type 2 diabetes mellitus, cardiovascular disease, and cancer (WHO, 2000). Underpinning the risk association between obesity and these diseases is the accumulation of excess adipose tissue that elicits an aberrant innate immune response causing local and systemic chronic inflammation, the hallmarks of which include increased pro‐inflammatory cytokine levels yielding increased production of free radicals, including reactive oxygen species (ROS) (Crujeiras *et al*., 2013; Grivennikov *et al*., 2010; Rocha and Libby, 2009; Wellen and Hotamisligil, 2005).

The number of cancer cases worldwide attributable to obesity is substantial and increasing (Kyrgiou *et al*., 2017; Massetti *et al*., 2017; Pearson‐Stuttard *et al*., 2018). It is becoming more common for younger individuals to be diagnosed with obesity‐related cancers (Massetti *et al*., 2017), and women bear a greater burden than men (Afshin *et al*., 2017; Massetti *et al*., 2017). Contributing to this burden is the association between obesity and breast cancer, the most common cancer in women (Ferlay *et al*., 2015). The association for obesity and diagnosis of estrogen receptor alpha (ERα)‐positive, hormone‐dependent breast cancer in postmenopausal patients was recognized early (Lipsett, 1975). A prevailing idea regarding the molecular mechanism is that ERα‐positive cancer in postmenopausal women is fueled by estrogens that are synthesized by adipose tissue in response to inflammatory signaling factors (Simpson and Brown, 2013). More recent epidemiological studies report that obesity is a risk factor for triple‐negative breast cancer (TNBC) diagnosis (Chen *et al*., 2016; Dolle *et al*., 2009; Gaudet *et al*., 2011; Gershuni *et al*., 2017; Phipps *et al*., 2011; Pierobon and Frankenfeld, 2013; Yang *et al*., 2011) and worse cancer‐associated outcomes (Berclaz *et al*., 2004; Ewertz *et al*., 2011; Fontanella *et al*., 2015; Hao *et al*., 2015; Liu *et al*., 2018). A TNBC diagnosis means that the tumor cancer cells lack expression of ERα, progesterone receptor, and the HER2 oncogene, a member of the epidermal growth factor family of receptor tyrosine kinases. Based on collective evidence that obesity‐induced chronic inflammation is a common factor promoting other diseases, we reasoned that inflammation also serves as the general link between obesity and TNBC. However, the exact molecular mechanism remains unknown.

We previously identified that ROS‐dependent expression of epigenetic reader methyl‐CpG‐binding domain protein 2 (MBD2), specifically the alternative mRNA splicing variant MBD2_v2, is crucial for maintenance and expansion of self‐renewing cancer stem cell‐like cells (CSCs) in TNBC cell cultures (Bao *et al*., 2017). Moreover, in heterogeneous cultures MBD2_v2 expression is contained in the CSC fraction (Bao *et al*., 2017). The relevance of CSCs is that they are a subpopulation of cancer cells recognized as the source of malignant tumor initiation (Chaffer and Weinberg, 2015; McDermott and Wicha, 2010; Reya *et al*., 2001), and they give rise to drug resistance and metastatic recurrence (Dave *et al*., 2012; Fillmore and Kuperwasser, 2008; Liu *et al*., 2010; Pattabiraman and Weinberg, 2014). Due to its function to maintain and promote expansion of tumor‐initiating CSCs, ROS‐dependent MBD2_v2 may be a key molecular feature driving TNBC incidence and recurrence. Considering that obesity is coupled with inflammation and ROS (Crujeiras *et al*., 2013), we hypothesized that obesity can fuel an increase in MBD2_v2 expression to promote the tumor‐initiating CSC phenotype in TNBC cells, setting a course to understanding why obesity is a risk factor for TNBC diagnosis and poor outcomes. Here, we report analysis of patient specimens and *in vivo* data supporting our hypothesis. Also, it was previously reported that serine‐ and arginine‐rich splicing factor 2 (SRSF2) is necessary for expression of MBD2_v2 in human pluripotent stem cells (hPSCs) (Lu *et al*., 2014). We present new mechanistic evidence that ROS‐dependent expression of SRSF2 drives TNBC MBD2_v2 expression and tumor‐initiating CSCs.

## 2 Materials and methods

### 2.1 Testing for associations between tumor MBD2_v2 expression levels and patient outcomes and body mass index (BMI)

Associations between relapse‐free survival and gene expression were determined for MBD2_v2 and SRSF2 using the Kaplan–Meier (KM) Plotter database for breast cancer (Gyorffy *et al*., 2010). The analysis was restricted to ERα‐, PR‐, and HER2‐negative tumors, that is, TNBC. For MBD2_v2, the transcript‐specific probe 214396_s_at (exon 3) was used to query data from 246 samples combined from five datasets, E‐MTAB‐365, GSE19615, GSE21653, GSE2603, and GSE31519. Auto select best cutoff and Censor at threshold options were selected. Quality control included removal of redundant samples and exclusion of outlier arrays. The same parameters and combined datasets were used in analysis of the full‐length isoform MBD2_v1 using MBD2_v1‐specific probe 202484_s_at (exon 6) and SRSF2 expression using the probe 200753_x_at, identified as being optimal by the JetSet best probe function (Li *et al*., 2011). Microarray gene expression data from a retrospective cohort of archived formalin‐fixed paraffin‐embedded (FFPE) tumors from 59 African American women diagnosed with TNBC at the Karmanos Cancer Institute (KCI) Detroit, MI, USA, between 2004 and 2010 were used to analyze the relationship between gene expression and body mass index (BMI). The approach to analyzing gene expression in de‐identified patient tumor specimens was prereviewed and approved by the Wayne State University Institutional Review Board. Written informed consent was obtained from all tissue donors, and methods conformed to the standards set by the Declaration of Helsinki. Gene expression data were generated using the GeneChip Human Gene 2.1 ST Array after amplification of RNA using the Affymetrix WT Pico Kit (Affymetrix, Santa Clara, CA, USA). Raw probe intensity data were normalized as implemented by the rma function in R to perform background subtraction, quantile normalization, and log2 transformation. Probe sets were not summarized to analyze alternative splice variants of our genes of interest. Linear regression analysis was performed, followed by a Student's *t*‐test (one‐sided) to measure significance of the mean increase for MBD2_v2 expression in tumors from patients with a BMI ≥ 30 (*n* = 28) relative to BMI < 30 (*n* = 31). Data are provided in Table S1.

### 2.2 Cell lines, culture conditions, and mammosphere formation assay

Triple‐negative breast cancer cell lines MDA‐MB‐468 and MDA‐MB‐231 were acquired from American Type Culture Collection (ATCC, Manassas, VA, USA) and routinely cultured in 10% FBS Dulbecco's modified Eagle's medium, at 37 °C, 5% CO_2_. The SUM149 TNBC cell line, developed by and acquired from Stephen Ethier (Forozan *et al*., 1999), was cultured in 5% FBS HAM F‐12 media containing 1 μg·mL^−1^ hydrocortisone and 5 μg·mL^−1^ human insulin. These three cell lines represent the TNBC molecular subtype. The cell lines were authenticated by short tandem repeat analysis using the PowerPlex(r) 16 System from Promega (Madison, WI, USA). The presence and self‐renewal capacity of CSCs were analyzed in TNBC cell cultures using the mammosphere formation assay, as performed previously (Bao *et al*., 2017), and described in Shaw *et al*. (2012). Mammospheres formed were counted and reported as a fraction of the total number of cells seeded (1000). Images were taken using a Eclipse TE2000‐U microscope (Nikon, Tokyo, Japan). The MAK164 Intracellular Assay (Sigma‐Aldrich, St. Louis, MO, USA) was used to measure hydrogen peroxide levels in cultured cells.

### 2.3 Semiquantitative RT‐PCR and immunoblot analysis

Real‐time, semiquantitative RT‐PCR analysis was performed as previously described (Bao *et al*., 2017; Teslow *et al*., 2018). RNA was harvested from pulverized snap‐frozen tumors surgically excised from euthanized mice or from cultured cell lines using the RNeasy kit from Qiagen (Valencia, CA, USA). RNA from tumors was additionally filtered using the OneStep PCR Inhibitor Removal kit (Zymo Research, Irvine, CA). The High‐Capacity RNA‐to‐cDNA Kit (Thermo Fisher Scientific, Waltham, MA, USA) was used to prepare cDNA. Reactions were run in triplicate using the StepOnePlus Real‐Time PCR System (Thermo Fisher Scientific). Relative expression was calculated by the delta–delta *C*_t_ method (Bookout *et al*., 2006). MBD2_v2 and RPLPO control gene were measured using TaqMan assays (Fisher Scientific, catalogue (cat.) number (no.) Hs00210557 and Hs99999902). PCR primers synthesized by IDT (Coralville, IA, USA) and FastStart SYBR Green Master Mix (Roche, Indianapolis, IN, USA) were used to analyze SRSF2 (PrimerbankID (Wang *et al*., 2012): 306482644c1), NANOG (PrimerbankID: 153945815c3), and β‐actin control gene (forward: CCCAGCACAATGAAGATCAA, reverse: ACATCTGCTGGAAGGTGGAC). Immunoblot analysis was performed as we have described previously (Bao *et al*., 2017). Briefly, protein lysates were harvested using the NE‐PER Nuclear and Cytoplasmic Extraction Kit (cat. no. 78833; Thermo Fisher Scientific) and concentrations measured by the Bradford assay. Nuclear protein samples (50 μg) were separated on a 10–12% SDS/PAGE gel and transferred to a nitrocellulose membrane (GE Healthcare, Pittsburgh, PA, USA). Primary antibodies targeted human MBD2 (cat no. A301‐633A‐M; Bethyl Laboratories, Montgomery, TX, USA), SRSF2 (cat. no. ab204916; Abcam, Cambridge, UK), and nucleoporin p62 (cat. no. 610497; BD Biosciences, Franklin Lakes, NJ, USA). Scanned films of full‐length blots are in Fig. S1.

### 2.4 Stable SRSF2 knockdown and MBD2_v2 overexpression in cell lines

Lentivirus‐mediated, shRNA knockdown of SRSF2 expression was done as previously described using the Open Biosystems Expression Arrest GIPZ lentiviral shRNAmir system (Bollig‐Fischer *et al*., 2011). MDA‐MB‐468 cells were transduced with vectors targeting SRSF2 (cat. no. RHS4430‐98485060 and RHS4430‐101104677) or the nonsilencing control vector. Stable overexpression of MBD2_v2 in MDA‐MB‐231 cells was performed as previously described (Bao *et al*., 2017). Packaged lentiviral particles to overexpress MBD2_v2 (NM015832.4) or GFP were purchased from Cyagen Biosciences (Santa Clara, CA, USA).

### 2.5 Animal work

All experiments and procedures involving animals and their care were prereviewed and approved by the Wayne State University Institutional Animal Care and Use Committee. Female B6.129S7‐Rag1tm1Mom/J (B6.Rag1−/−) mice were purchased from Jackson Laboratory (cat. no. 002216, Bar Harbor, ME, USA) at 5 weeks old and were acclimated for 1 week on standard chow diet. After 1 week, all mice were switched to and thereafter maintained on a purified diet. For experiments assessing tumor formation without consideration of diet‐induced obesity (DIO), mice were fed a control formula (kcal fat% = 10, gram% = 4.3) from Research Diets (cat. no. D12450B, New Brunswick, NJ, USA). To study the effects of DIO, groups of mice were randomized to receive the control formula diet or a high‐fat matched formula (kcal fat% = 60, gram% = 35, Research Diets, cat. no. D12492). At 11 weeks old (5 weeks of formula diet), mice were aseptically inoculated with cancer cells, in the flank, subcutaneously by injection using a 1‐cc TB syringe with a 25‐g half‐inch needle, in a volume of 0.1–0.25 mL with Matrigel (1 : 1 ratio). Resulting tumor mass (mg) was calculated based on caliper measurements [tumor mass = (*l*·*w*2)/2]. In experiments assessing effects of DIO on tumor formation, mice were inoculated with 10^6^ titers of MDA‐MB‐231 cells (bilaterally) or MDA‐MB‐468 cells (unilaterally). For these experiments, there were six inoculations of MDA‐MB‐231 in control mice (three mice) and eight inoculations in DIO mice (four mice). For the MDA‐MB‐468 cell line, there were six inoculations per group (12 total mice). To compare MBD2_v2‐overexpressing and GFP‐expressing MDA‐MB‐231 cell lines, mice were unilaterally inoculated with a 10^5^ cell titer, using six mice per engineered cell line (12 total mice). To compare tumor formation by SRSF2 knockdown and nonsilencing shRNA control MDA‐MB‐468 cells, DIO mice were unilaterally inoculated with 10^5^ titer, using six mice per engineered cell line (12 total mice). To assess DIO‐induced oxidative stress, malondialdehyde (MDA) levels were measured in liver tissues aseptically harvested from randomly selected DIO and control mice (six per group) using the Lipid Peroxidation (MDA) Assay Kit (Sigma‐Aldrich, St. Louis, MO, USA), following instructions for colorimetric detection.

### 2.6 Genomewide expression profiling of tumors harvested from mice

RNA was isolated from MDA‐MB‐468 cell line‐derived tumors harvested from lean control mice (*n* = 3) and DIO mice (*n* = 3). Genomewide expression was measured using the SurePrint G3 Human Gene Expression 8 × 60 K Microarray and Low Input Quick Amp Labeling kits (Agilent Technologies, Santa Clara, CA, USA). Arrays were scanned on the SureScan Microarray Scanner System (Agilent Technologies). Data extraction was performed using feature extraction software (Agilent Technologies). The limma package in r (https://www.r-project.org/) was used to perform normal–exponential convolution (with an offset of 50) background correction; loess normalization within arrays; and quantile normalization across arrays. The difference in gene expression (per gene) was assessed by adjusting for multiple probes per gene, unequal variance within groups, and correlated observations within the generalized least squares model framework. The resulting set of significant (*P* ≤ 0.01) differentially expressed genes (RefSeq coding IDs) were analyzed for further significance according to pathway overenrichment analysis using the Enrichr tool and NCI‐Nature Pathways library (Chen *et al*., 2013). The *P* value of overlap and the adjusted enrichment value (Enrichment Score), optimized for Enrichr (Kuleshov *et al*., 2016), are reported for top‐ranked pathways. The dataset is available through Gene Expression Omnibus accession number GSE114604.

### 2.7 Statistical analysis

Statistical analysis was performed using r 3.3.2 (http://www.R-project.org/). Graphs were generated with r 3.3.2 or graphpad prism (Graphpad Software, San Diego, CA, USA). *P* values ≤ 0.05 are reported as significant. Welch's *t*‐test was applied to semiquantitative RT‐PCR and mammosphere assay data. Gray's test of difference in cumulative incidence was used to assess the significance of differences observed in mouse tumor formation frequency and time to event between experimental groups. Fisher's exact test was used to assess the difference in tumor formation frequency on a given date. Generalized least squares, allowing for correlated observations in the same animal when appropriate (i.e., bilateral inoculation), assessing the interaction between group and time was utilized to test whether tumor growth rates were different between the groups under study. The mean body weight for mice was calculated for four cages in each condition, up to 6 mice per cage, measured 2–3 times per week for up to 185 days. A quadratic model was built to model the longitudinal data. Differences between groups at a given time point were compared using the linear predictor from the model and a Student's *t*‐test.

## 3 Results

### 3.1 Associations between tumor MBD2_v2 expression and patient outcomes and BMI

**Figure 1:**
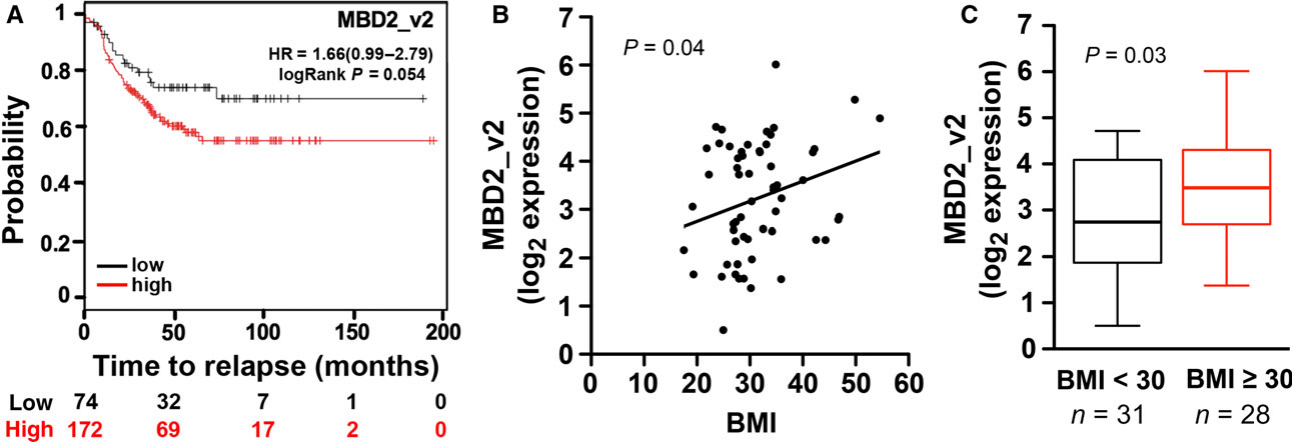
Associations between expression of MBD2_v2 in TNBC patient tumor specimens and survival outcomes and BMI. (A) Analysis was performed with the online KM Plotter database, using a logrank test of association between relapse‐free survival and MBD2_v2 transcript level. The number of subjects at risk at different time points is indicated below the *x*‐axis. Testing for gene transcript level associations with BMI was done using a separate gene expression microarray dataset generated from TNBC specimens collected at the KCI, where BMI data corresponding to de‐identified samples were available. (B) The association between BMI and MBD2_v2 expression was tested using linear regression analysis (*P* = 0.04, correlation 0.27). (C) The mean MBD2_v2 expression for tumors from obese patients with BMI ≥ 30 was compared to the mean MBD2_v2 expression for tumors from nonobese patients with BMI < 30. Line is equal to the median value (BMI < 30 median = 2.7, BMI ≥ 30 median = 3.5). The *P* value was calculated using a Student's *t*‐test (one‐sided). The number of patients (*n*) per group is indicated.

We hypothesized that obesity can cause an increase in MBD2_v2 expression to promote the tumor‐initiating CSC phenotype in TNBC cells, setting a course to understanding why obesity is a risk factor for TNBC diagnosis and poor outcomes. To establish the plausibility of our hypothesis, it was a priority to address the question: Do MBD2_v2 levels in TNBC patient tumor specimens associate with survival outcomes and BMI? Analysis using the KM Plotter database (Gyorffy *et al*., 2010), testing for associations between gene transcript levels and relapse‐free survival among 246 specimens, showed that high expression of MBD2_v2 in TNBC patient tumors associates with high rates of relapse [hazard ratio (HR) = 1.66, *P *=* *0.05, Fig. 1A]. The KM Plotter database lacks BMI data. To test for a relationship between patient BMI and tumor transcript expression, we used another existing probe‐based gene expression dataset comprising 59 TNBC specimens with known BMI status collected at the KCI (Table S1). Linear regression analysis indicated that there is a positive association for MBD2_v2 expression and BMI (*P *=* *0.04, correlation 0.27, Fig. 1B), and MBD2_v2 expression levels are significantly increased in tumors from patients with BMI ≥ 30 compared to tumors from patients with BMI < 30 (*P *=* *0.03, Fig. 1C). Based on similar analysis of these data sets, there is no association between tumor expression of the full‐length isoform MBD2_v1 and patient BMI, and high MBD2_v1 expression is associated with low rates of relapse (HR = 0.68, *P *=* *0.04, Fig. S2). The KCI dataset currently lacks a sufficient number of events to test for associations with outcomes.

### 3.2 Increased tumor formation frequency and tumor MBD2_v2 expression in DIO mice

**Figure 2:**
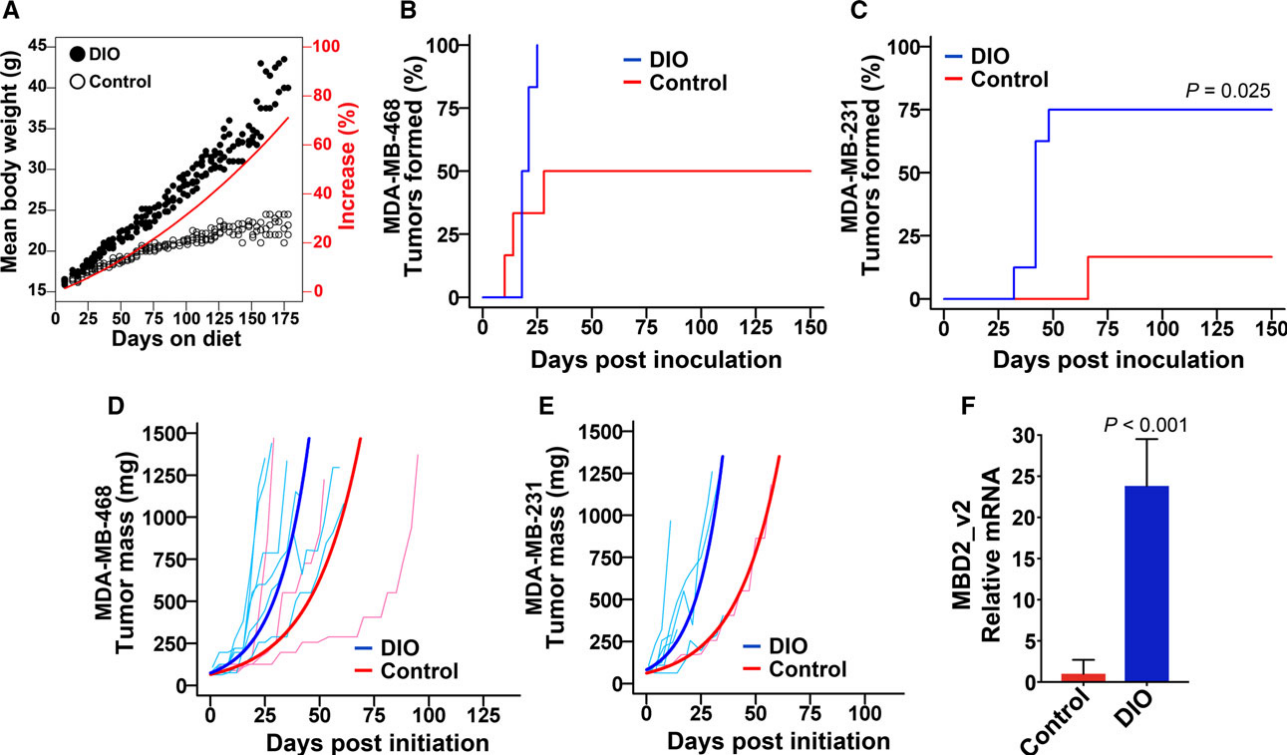
Tumor formation frequency and tumor MBD2_v2 expression are increased in DIO mice. (A) The mean body weight over time for DIO mice on the high‐fat, calorie‐dense formula diet and mice on control formula diet. On day 35, the weight increase in DIO mice was 10%, *P *<* *0.001. (B) MDA‐MB‐468 or (C) MDA‐MB‐231 TNBC cells were used to inoculate control and DIO female mice by subcutaneous flank injection. From time of inoculation, tumor formation frequency and time to initiation were measured over 150 days. The *P* values were calculated using Gray's test. *P* values ≤ 0.05 are reported. (D) MDA‐MB‐468 and (E) MDA‐MB‐231 tumor mass was plotted for all tumors formed with modeled growth (bold) superimposed. A generalized least squares test was used to calculate *P* values (*P *>* *0.05). (F) Semiquantitative RT‐PCR analysis was performed to measure MBD2_v2 transcript levels in RNA harvested from MDA‐MB‐468 tumors, comparing tumors harvested from DIO mice (*n* = 3 randomly selected) to those from control mice (*n* = 3). Data are expressed as the relative means ± SEM; Welch's *t*‐test was used to calculate the *P* value.

We investigated whether obesity causes increased TNBC cell tumor initiation capacity and increased tumor MBD2_v2 expression using female B6.129S7‐Rag1tm1Mom/J (B6.Rag1−/−) mice as a model for DIO. Due to a homozygous *Rag1* gene deletion, this model lacks mature T and B lymphocytes (Mombaerts *et al*., 1992); therefore, it can be used for human tumor xenograft and cancer cell implant studies (O'Neill *et al*., 2016; Ray *et al*., 2007; Zaytouni *et al*., 2017). The B6.Rag1−/− model does, however, maintain macrophages with the capacity to recapitulate the pro‐inflammatory environment and oxidative stress induced by increased adiposity (Lee *et al*., 2011), and like its C57BL/6J background—the mouse strain most commonly used to study cancer and obesity (Donohoe *et al*., 2017)—B6.Rag1−/− presents a DIO phenotype that mimics human obesity (Boden *et al*., 2015; Collins *et al*., 2004; Lee *et al*., 2011; O'Neill *et al*., 2016; Zaytouni *et al*., 2017). We employed two TNBC cell lines, MDA‐MB‐231 and MDA‐MB‐468, and began by assessing the impact of obesity on tumor formation rate. Groups of mice were randomly assigned either a control purified diet (kcal% = 10, gram% = 4.3) or a matched formula calorie‐dense, high‐fat diet (kcal% = 60, gram% = 35). As was reported previously for female C57BL/6J and B6.Rag1−/− mice (Nishikawa *et al*., 2007; Zaytouni *et al*., 2017), by day 35 the mice on the high‐fat diet exhibited a significant weight increase relative to control mice (*P <* 0.001, Fig. 2A). On day 36, groups of DIO mice and lean controls were inoculated with MDA‐MB‐231 or MDA‐MB‐468 cells. Mice were monitored for tumor formation up to 150 days postinoculation, and tumor formation frequencies were calculated. Relative to control mice, the tumor formation frequency for DIO mice was increased twofold for the MDA‐MB‐468 cell line (Fig. 2B) and approximately fourfold for the MDA‐MB‐231 cell line (*P *=* *0.025, Fig. 2C). The rates of MDA‐MB‐468 cell line tumor formation in each condition, control and DIO mice, were greater than the rates for MDA‐MB‐231 cells. MDA‐MB‐468 cultures, prior to inoculation, also expressed higher endogenous levels of MBD2_v2 relative to MDA‐MB‐231 cells (Fig. S3).

These experiments were devised to compare tumor formation rates, but tumor mass was plotted (Fig. 2D,E). The upward slopes of the growth curves are similar, indicating that DIO had little or no effect on the growth rates of established MDA‐MB‐468 or MDA‐MB‐231 tumors. We performed semiquantitative RT‐PCR analysis of tumor MBD2_v2 expression. MBD2_v2 levels were higher in tumors harvested from DIO mice compared to tumors harvested from control mice (*P <* 0.001, Fig. 2F).

### 3.3 Increasing MBD2_v2 expression in TNBC cells increases tumor initiation capacity

**Figure 3:**
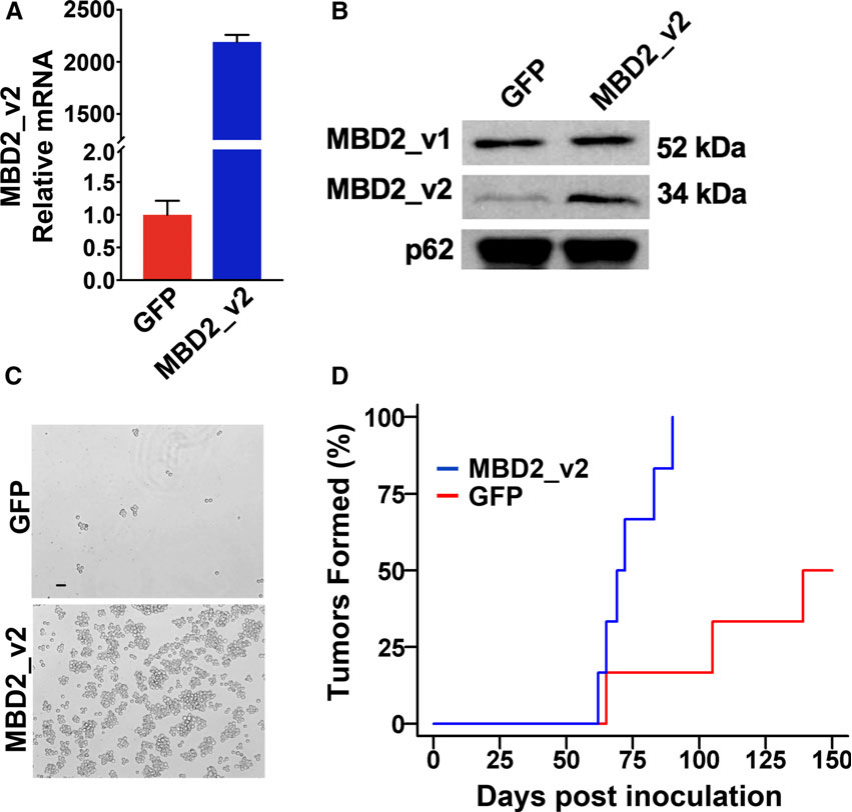
MBD2_v2 overexpression in TNBC cells increases *in vivo* tumor initiation capacity. (A) Stable overexpression of MBD2_v2 isoform in the MDA‐MB‐231 cell line was confirmed by semiquantitative RT‐PCR analysis (relative means ± SD of three technical replicates); (B) and by immunoblot analysis of nuclear lysates, with nucleoporin p62 serving as the loading control. (C) MBD2_v2‐overexpressing MDA‐MB‐231 cells or GFP‐expressing control MDA‐MB‐231 cells were seeded equally under serum‐free nonadherent conditions in a mammosphere formation assay. Images documenting the differences in numbers of spheres formed were taken after 7 days. Bar = 50 μm, 4× magnification. (D) MBD2_v2‐overexpressing or GFP‐expressing MDA‐MB‐231 cells were subcutaneously inoculated by injection into the flank regions of mice, *n* = 6 per group. At day 100, the difference in tumor formation frequency was calculated (*P* = 0.02, Fisher's exact test). At 150 days postinoculation, the difference in cumulative incidence was also assessed (*P* = 0.12, Gray's test).

To more directly test whether increased MBD2_v2 causes increased tumor initiation capacity, we stably overexpressed MBD2_v2 in TNBC cells prior to inoculation. We proceeded to re‐establish, as recently reported using other TNBC lines (Bao *et al*., 2017), that MBD2_v2 overexpression promotes expansion of the CSC fraction in MDA‐MB‐231 TNBC cell cultures using a mammosphere formation assay. Stable overexpression of MBD2_v2 in cells by lentiviral transduction, confirmed by immunoblot and semiquantitative RT‐PCR analysis (Fig. 3A,B), caused a marked increase in the numbers of mammospheres that grew from equal seeding under nonattachment serum‐free culture conditions relative to a stable GFP‐expressing MDA‐MB‐231 control cell line (Fig. 3C). We inoculated mice with MBD2_v2‐overexpressing or GFP‐expressing MDA‐MB‐231 cells. By day 100, six of six mice inoculated with MBD2_v2‐overexpressing MDA‐MB‐231 cells bore tumors, yet at the same time, only one of six mice carried tumors in the GFP control group (Fig. 3D). The experiment was extended to 150 days postinoculation; at which point, three of six mice remained tumor‐free in the GFP control group (Fig. 3D). Tumor mass was documented over the course of the experiment, and according to growth curve plots, MBD2_v2 overexpression did not affect the rate of tumor growth (Fig. S4). This is consistent with the insight that MBD2_v2 promotes CSCs, which are not highly proliferative (Pattabiraman and Weinberg, 2014).

### 3.4 TNBC cell MBD2_v2 expression depends on antioxidant‐sensitive SRSF2 expression

**Figure 4:**
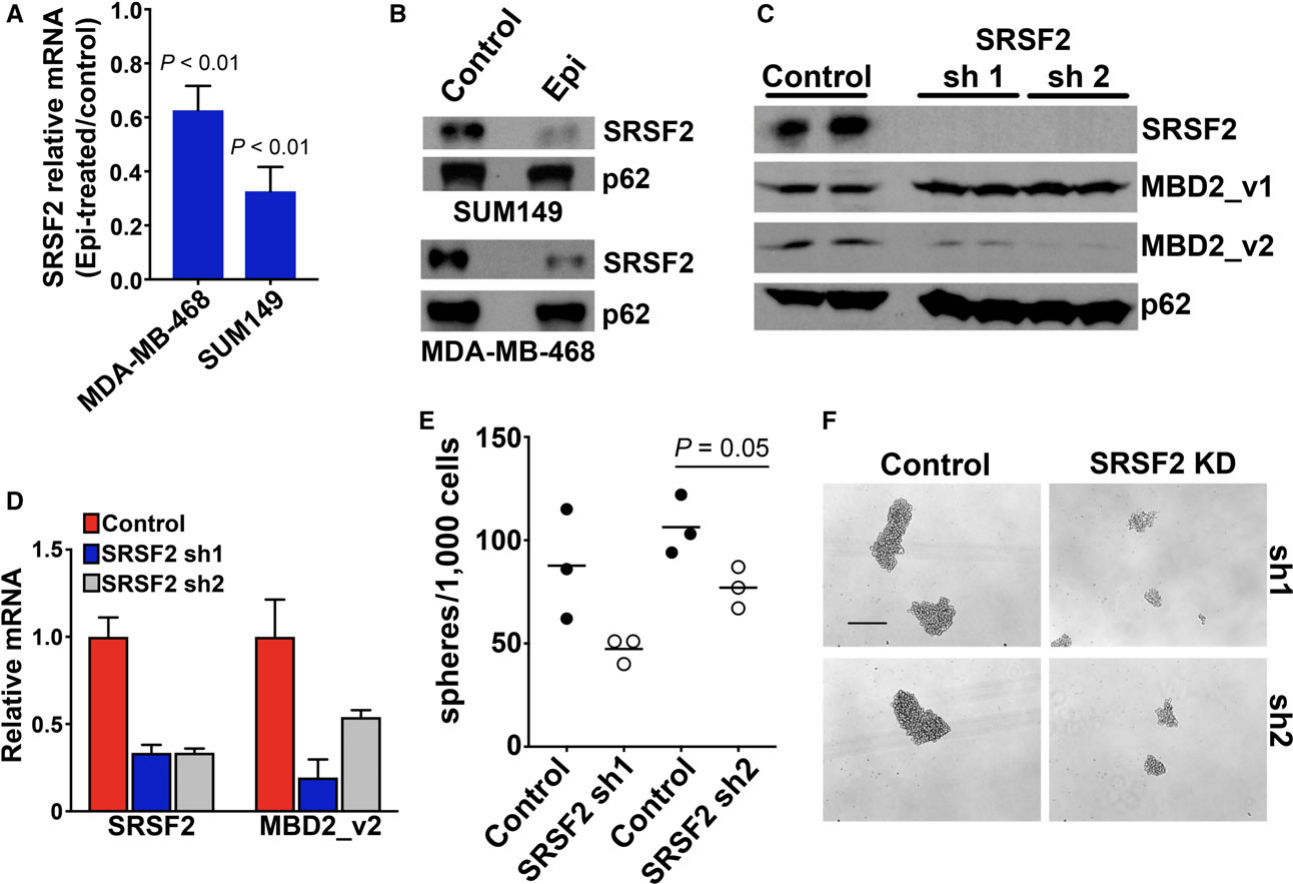
Knockdown of SRSF2 decreases MBD2_v2 levels in TNBC cells. (A) The effect of (–)‐epicatechin (Epi) antioxidant treatment (48 h, 120 μm) to reduce readily detectable SRSF2 levels in MDA‐MB‐468 and SUM149 TNBC cell lines was measured by semiquantitative RT‐PCR analysis of RNA (B) and by immunoblot analysis of protein lysates. Semiquantitative RT‐PCR (mean fold‐change for sets of three technical replicates ± SD) and immunoblot data are representative of two independent experiments for each cell line. (C) Confirmation of stable knockdown of SRSF2 in MDA‐MB‐468 cells and the impact of SRSF2 knockdown on MBD2_v2 levels were measured by semiquantitative RT‐PCR analysis (relative means of three technical replicates ± SD) (D) and by immunoblot analysis. (E) A mammosphere formation assay was used to simultaneously observe the impact of SRSF2 knockdown by each shRNA construct on the numbers of mammospheres (F) and the size of mammospheres formed. Images and counts were taken 7 days after passaging to serum‐free, nonadherent conditions. Results in (E) and (F) are one complete set of data and are representative of two independent experiments. Bar = 500 μm, 4× magnification. Welch's *t*‐test was applied to semiquantitative RT‐PCR and mammosphere assay data.

It is reported that splicing factor SRSF2 is necessary for expression of alternative mRNA splicing variant MBD2_v2 in human pluripotent stem cells (hPSCs) (Lu *et al*., 2014). We designed a set of experiments to examine whether the same regulatory relationship between SRSF2 and MBD2_v2 exists in TNBC cells. First, we observed that expression of SRSF2 is, like MBD2_v2 (Bao *et al*., 2017), subject to antioxidant‐sensitive, ROS‐regulation in TNBC cells. Using MDA‐MB‐468 and SUM149 TNBC cell lines, which expressed similarly abundant endogenous levels of SRSF2, (–)‐epicatechin antioxidant treatment reduced ROS and MBD2_v2 levels (Fig. S5) and downregulated SRSF2 mRNA and protein levels (Fig. 4A,B). We then established two independent SRSF2 stable knockdown (using two unique shRNA sequences) and nonsilencing vector control MDA‐MB‐468 cell lines. The knockdown of SRSF2 resulted in decreased MBD2_v2 protein and mRNA levels (Fig. 4C,D). According to mammosphere formation assays, SRSF2 knockdown also resulted in fewer mammospheres (Fig. 4E), and a reduction in size of those that did survive (Fig. 4F). Altogether, this characterizes a role for the ROS‐dependent SRSF2–MBD2_v2 regulatory axis in TNBC cells.

### 3.5 Tumor SRSF2 expression is increased in DIO mice, and downregulation of SRSF2 hinders tumor initiation capacity of TNBC cells

**Figure 5:**
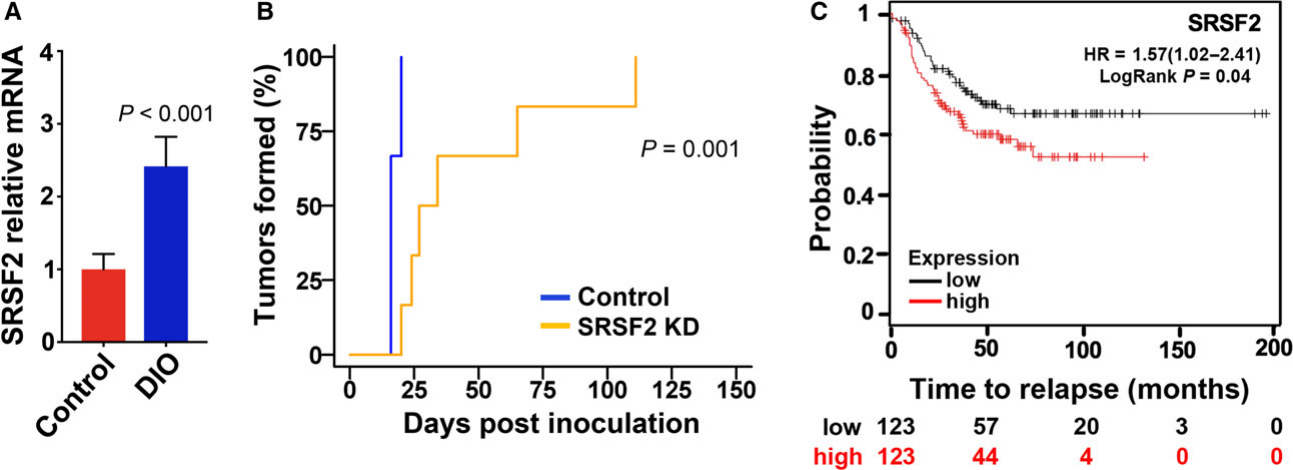
Tumor SRSF2 expression is increased in DIO mice, and downregulation of SRSF2 hinders tumor formation. (A) Comparison of SRSF2 levels in wild‐type MDA‐MB‐468 tumors harvested from DIO mice (*n* = 3 randomly selected) and lean control mice (*n* = 3). Relative means ± SEM, *P* value Welch's *t*‐test. (B) SRSF2 knockdown and nonsilencing vector control MDA‐MB‐468 cells were subcutaneously inoculated by injection into the flank regions of mice, *n* = 6 per group. Gray's test of difference in cumulative incidence was used to calculate the *P* value. (C) Analysis was performed with the online KM Plotter database, using a logrank test of associations between relapse‐free survival and SRSF2 transcript levels. The number of subjects at risk at different time points is indicated below the *x*‐axis.

We performed semiquantitative RT‐PCR analysis for SRSF2 expression in tumors harvested from DIO and control mice. Like MBD2_v2 (Fig. 2F), SRSF2 levels were consistently higher in tumors harvested from DIO mice (*P <* 0.001, Fig. 5A). To more directly assess whether increased SRSF2 has a role in increased tumor initiation capacity, we selected one of the stable knockdown cell lines (SRSF2 sh2) to test whether downregulating SRSF2 yields decreased tumor initiation capacity in the high tumorigenic context of MDA‐MB‐468 cells in DIO mice. SRSF2 knockdown cells demonstrated significantly delayed tumor initiation relative to nonsilencing control cells (*P < *0.05, Fig. 5B). By day 24 postinoculation, tumors were formed in 100% of mice inoculated with nonsilencing control cells (six of six), and at the same time point, only 33% of mice (two of six) bore tumors in the SRSF2 knockdown group (Fig. 5B). Tumor mass was also documented over the course of the experiment, and there was no significant difference in growth rates comparing SRSF2 knockdown and control tumors (Fig. S6). This remains consistent with insight that SRSF2–MBD2_v2 promotes CSCs, which are not highly proliferative (Pattabiraman and Weinberg, 2014). According to semiquantitative RT‐PCR analysis, SRSF2 knockdown was lost in established tumors (Fig. S6). In addition, high expression of SRSF2 in TNBC patient tumors associates with high rates of relapse (HR = 1.57, *P *=* *0.04, KM Plotter database, Fig. 5C). However, analysis of the KCI dataset, which revealed an association between MBD2_v2 and BMI (Fig. 1B,C), failed to identify an association between SRSF2 expression and BMI (Fig. S6).

## 4 Discussion

Triple‐negative breast cancer is a molecular subtype that accounts for 15% of invasive breast cancer diagnoses (Brewster *et al*., 2014; Thakur *et al*., 2018). Incidence rates in developing countries and among women of African ancestry are higher (Brewster *et al*., 2014; Thakur *et al*., 2018). TNBC is also more prevalent in younger, premenopausal women (Bauer *et al*., 2007; Carey *et al*., 2006), and obesity is a risk factor for TNBC diagnosis (Chen *et al*., 2016; Dolle *et al*., 2009; Gaudet *et al*., 2011; Gershuni *et al*., 2017; Phipps *et al*., 2011; Pierobon and Frankenfeld, 2013; Yang *et al*., 2011) and worse cancer‐associated outcomes (Berclaz *et al*., 2004; Ewertz *et al*., 2011; Fontanella *et al*., 2015; Hao *et al*., 2015; Liu *et al*., 2018). Ultimately, women diagnosed with TNBC have the lowest 5‐year survival rates among all breast cancer patients, in large part due to a lack of therapeutic options (Bianchini *et al*., 2016). For TNBC, the molecular drivers remain uncertain and targeted therapies do not exist. Moreover, development of transformative treatment strategies for TNBC must first identify and then find a way to target factors driving the tumor‐initiating CSCs, which also give rise to drug resistance and metastatic recurrence (Dave *et al*., 2012; Fillmore and Kuperwasser, 2008; Liu *et al*., 2018; Pattabiraman and Weinberg, 2014). These clinical challenges further underscore the value of our investigation.

Based on a review of the literature, we have generated the earliest reports on the role of epigenetic reader and alternative mRNA splicing variant MBD2_v2 to sustain and promote the tumor‐initiating CSC phenotype, first based on studies conducted *in vitro* (Bao *et al*., 2017; Teslow *et al*., 2018), and now based on *in vivo* experiments that link it to obesity. The results herein also elucidate that splicing factor SRSF2 is necessary for expression of MBD2_v2 in TNBC cells and for CSC survival. Moreover, SRSF2 and MBD2_v2 expression in TNBC cells is dependent on antioxidant‐sensitive ROS. We investigated whether obesity impacts SRSF2 and MBD2_v2 by inoculating a DIO mouse model with tumor‐forming TNBC cell lines, and in agreement with our hypothesis, SRSF2 and MBD2_v2 expression levels were significantly upregulated in tumors harvested from DIO mice displaying increased tumor formation rates. The DIO mice readily exhibited increased visceral adiposity, and we verified that systemic oxidative stress levels were increased in DIO mice relative to control mice by measuring liver MDA, a lipid peroxidation marker (Vincent and Taylor, 2006) (Fig. S7), but a possible shortcoming of our study is that we did not attempt to treat DIO mice systemically with (–)‐epicatechin antioxidant in order to affirm that inflammation, ROS specifically, was regulating increased SRSF2 and MBD2_v2 expression in TNBC cell line‐derived tumors as in TNBC cell line cultures (Fig. 4A) (Bao *et al*., 2017). However, it is well‐documented in the literature that dysfunctional adipose tissue and resident macrophages function as an endocrine organ, producing pro‐inflammatory cytokines that directly act on tumors (van Kruijsdijk *et al*., 2009), and more detailed insights for how the B6.Rag1−/− DIO mouse model system likely parallels human physiology in this regard came to light when we applied genomewide analysis to evaluate the greater impact of DIO on TNBC tumor gene expression. The significant results highlighted evidence of the effects of pro‐inflammatory cytokines, specifically interferon‐gamma (IFNγ) signaling (Fig. S7). Circulating IFNγ is produced by adipocytes in obese individuals, and IFNγ levels are elevated by DIO in B6.Rag1−/− mice (Liu *et al*., 2015; Wentworth *et al*., 2017). Activation of breast cancer cell IFNγ receptors increases ROS levels, specifically hydrogen peroxide (Zibara *et al*., 2017). Also in obese breast cancer patients, increased macrophage infiltration of breast adipose tissue yields additional paracrine‐acting pro‐inflammatory cytokines (Vaysse *et al*., 2017).

For experiments designed to more directly assess whether increased expression of MBD2_v2 and SRSF2 plays a causative role in increased tumor formation, we stably modified the levels of MBD2_v2 or SRSF2 in TNBC cells prior to inoculation. MBD2_v2 overexpression significantly increased tumor initiation capacity of TNBC cells in lean mice, and SRSF2 knockdown, which decreased MBD2_v2 expression, significantly hindered tumor formation capacity in the more tumorigenic context of DIO mice. The relevance of the experimental methodology to inoculate mice with cancer cells to measure efficiency of tumor formation, or tumorigenicity, was previously established (Al‐Hajj *et al*., 2003). Researchers observed that relatively small numbers of cells exhibiting the CSC phenotype possess the capacity to macrocolonize and subsequently form tumors in mice, but greater numbers of cells with alternate phenotypes, referred to as bulk cancer cells, fail to macrocolonize (Al‐Hajj *et al*., 2003).

The *in vitro* and *in vivo* experimental data presented here support that the SRSF2–MBD2_v2 regulatory axis is a feature necessary for maintenance of TNBC tumor‐initiating CSCs that can be induced to expand the CSC fraction. Therefore, SRSF2–MBD2_v2 expression would not be exclusive to, but increased in TNBC tumors from obese patients and patients with poor survival outcomes. Results from our analysis of patient tumor sample data are in‐line with this idea. KM Plotter database inquiries revealed that high mRNA expression of MBD2_v2 and SRSF2 in TNBC specimens associates with high rates of relapse. MBD2_v2 levels also positively associate with BMI and are significantly higher in tumors from obese women; however, the same dataset did not show an association for SRSF2 and BMI. This does not necessarily contradict our mechanistic evidence that MBD2_v2 expression in TNBC cells depends on SRSF2; it may reflect that differences in SRSF2 mRNA levels are small and challenging to discern in analysis of RNA from patient FFPE cancer specimens. This rationale is in‐line with results from our analysis of tumors harvested from DIO mice relative to lean controls: SRSF2 expression was increased four‐fold in DIO tumors, and its target, MBD2_v2 expression, was increased 20‐fold.

The function of MBD2_v2 to regulate TNBC CSCs is underscored by the necessity for MBD2_v2 to maintain the self‐renewing capacity of human pluripotent stem cells (hPSCs) (Lu *et al*., 2014). A report by Lu *et al*. (2014) details the mechanism whereby MBD2_v2 activates essential hPSC factors such as NANOG. We also observed that increasing MBD2_v2 upregulates stem cell marker NANOG expression in TNBC cells, and NANOG levels are increased in tumors harvested from DIO mice relative to tumors from control mice (Fig. S8). Moving forward, we will continue work to elucidate the mechanistic pathway leading to aberrant, upregulated MBD2_v2 expression dependent on ROS and likely subject to inflammation related to obesity. Another priority is to study whether SRSF2 and MBD2_v2 play a role in malignant transformation of partly transformed or noncancerous breast epithelial cells and whether this too may be induced by obesity. It is notable that the dataset used to uncover the positive association between MBD2_v2 expression and BMI consists entirely of specimens from African American women. African American women are approximately two times more often obese relative to European American women (Yang and Colditz, 2015), and a TNBC driver mechanism fueled by obesity could contribute to the worse TNBC outcomes and higher incidence of TNBC among African American women (Brewster *et al*., 2014; Dietze *et al*., 2015). We expect the association between MBD2_v2 and BMI to be similar irrespective of race, yet it is possible that planned analysis of tumors from European American women will not so readily demonstrate the association. Conversely, lifestyle factors contributing to systemic inflammation independent of obesity such as sleep deprivation and psychosocial stress are also more prominent in African Americans (Brody *et al*., 2015; Carroll *et al*., 2009; Curtis *et al*., 2017; Vgontzas *et al*., 1999), and inflammation in nonobese patients may influence the SRSF2‐MBD2_v2 axis and be a confounding variable for the association between obesity and MBD2_v2 expression in tumors from African American women. Either way, our study provides a new avenue for research to understand the molecular biology of race‐associated TNBC disparities.

Finally, in our retrospective analysis of MBD2_v2 expression in patient tumor data we used the threshold BMI ≥ 30 kg·m^−2^ to define obesity (WHO, 2000). Obesity is a medical condition that applies to overweight individuals with excess visceral adiposity (WHO, 2000). While it is applicable to estimate obesity in the general population, a BMI calculation does not measure adipose tissue, nor does it inform an individual of their body fat distribution. Calculating the waist‐to‐hip ratio or more directly measuring body fat percentage is likely to provide more accurate assessments of patient adiposity, and results from research employing alternate approaches are raising awareness that the use of BMI and the BMI ≥ 30 kg·m^−2^ threshold to define obesity is underestimating rates of obesity and the impact of obesity on patients (Hamer *et al*., 2017; Rahman and Berenson, 2010). However, these types of data are not standard clinical information and were not available for our analysis. Moreover, the data used to calculate BMI (weight and height) are routinely available and the threshold BMI ≥ 30 kg·m^−2^ proved useful to establish that obesity is an adverse risk factor for TNBC (Chen *et al*., 2016; Dolle *et al*., 2009; Gershuni *et al*., 2017; Phipps *et al*., 2011; Pierobon and Frankenfeld, 2013; Yang *et al*., 2011).

## 5 Conclusion

The current report describes evidence to support that MBD2_v2 expression is responsive to obesity and drives TNBC tumorigenicity, and thus provides molecular insights into support of the epidemiological evidence that obesity is a risk factor for TNBC. The majority of TNBC patients are obese (Trivers *et al*., 2009; Vona‐Davis *et al*., 2008) and rising obesity rates threaten to further increase the burden of obesity‐linked cancers (Pearson‐Stuttard *et al*., 2018), which reinforces the relevance of this area of study.

## Conflict of interest

The authors declare no conflict of interest.

## Author contributions

ABF conceived of and oversaw the study. EAT and BB performed molecular and cellular experiments. LAP supervised animal experiments and care. KSP generated human specimen data. EAT, CM, GD, and ABF analyzed data. LAP, RMM, GD, KSP, and ABF provided expertise and essential materials. EAT and ABF wrote the manuscript with feedback from all authors. All authors read and approved the final manuscript.

## Supporting information


**Fig. S1.** Scanned films of full‐length blots.
**Fig. S2.** Analysis of MBD2_v1 expression in patient tumors.
**Fig. S3.** MBD2_v2 expression in TNBC cell line cultures prior to mouse inoculation.
**Fig. S4.** MBD2_v2 overexpressing and GFP expressing tumor growth curves.
**Fig. S5.** Effect of (–)‐epicatechin treatment on ROS and MBD2_v2 levels in TNBC cell cultures.
**Fig. S6.** SRSF2 knockdown tumor levels and growth curves, and patient tumor SRSF2 expression related to BMI.
**Fig. S7.** Visceral adiposity, oxidative stress levels and enrichment of signaling pathway genes in tumors comparing DIO and control mice.
**Fig. S8.** NANOG in TNBC cell line cultures and tumors.
**Table S1.** Patient tumor gene expression and BMI data.
