# Supplementary material for: Obesity‐induced MBD2_v2 expression promotes tumor‐initiating triple‐negative breast cancer stem cells
Source: Mol Oncol. 2019 Mar 1;13(4):894–908. doi: 10.1002/1878-0261.12444 (PMC6441886; doi:10.1002/1878-0261.12444)
Supplement: Supplementary file 1 — Fig. S1. Scanned films of full‐length blots. Fig. S2. Analysis of MBD2_v1 expression in patient tumors. Fig. S3. MBD2_v2 expression in TNBC cell line cultures prior to mouse inoculation. Fig. S4. MBD2_v2 overexpressing and GFP expressing tumor growth curves. Fig. S5. Effect of (–)‐epicatechin treatment on ROS and MBD2_v2 levels in TNBC cell cultures. Fig. S6. SRSF2 knockdown tumor levels and growth curves, and patient tumor SRSF2 expression related to BMI. Fig. S7. Visceral adiposity, oxidative stress levels and enrichment of signaling pathway genes in tumors comparing DIO and control mice. Fig. S8. NANOG in TNBC cell line cultures and tumors. Table S1. Patient tumor gene expression and BMI data. [file MOL2-13-894-s001.pdf]

Supporting Information

Obesity-induced MBD2_v2 expression promotes tumor-initiating triple negative breast cancer stem cells

Emily A. Teslow, Cristina Mitrea, Bin Bao, Ramzi M. Mohammad, Lisa A. Polin, Greg Dyson, Kristen S. Purrington and Aliccia Bollig-Fischer

Description of Additional Files:

Table S1. Patient tumor gene expression and BMI data.

Fig. S1. Scanned films of full-length blots.

Fig. S2. Analysis of MBD2_v1 expression in patient tumors.

Fig. S3. MBD2_v2 expression in TNBC cell line cultures prior to mouse inoculation.

Fig. S4. MBD2_v2 overexpressing and GFP expressing tumor growth curves.

Fig. S5. Effect of (–)-epicatechin treatment on ROS and MBD2_v2 levels in TNBC cell cultures.

Fig. S6. SRSF2 knockdown tumor levels and growth curves, and patient tumor SRSF2 expression related to BMI.

Fig. S7. Visceral adiposity, oxidative stress levels and enrichment of signaling pathway genes in tumors comparing DIO and control mice.

Fig. S8. NANOG in TNBC cell line cultures and tumors.

Table S1. Expression levels of MBD2 and SRSF2 transcripts in TNBC patient tumor samples.

SAMPLE_ID	BMI	MBD2_v1	MBD2_v2	SRSF2_v1	SRSF2_v2
1	42.19	5.040	4.250	2.760	3.200
2	33.91	4.070	4.550	2.570	3.600
3	34.45	4.170	3.460	2.520	4.030
4	24.69	4.250	4.660	3.000	3.700
5	46.69	2.460	2.790	1.030	3.140
6	33.97	3.080	3.890	1.000	3.080
7	34.45	3.950	3.400	1.240	3.680
8	21.82	4.940	4.270	1.470	3.600
9	28.6	2.980	4.110	1.580	3.480
10	27.65	2.180	1.860	1.170	2.120
11	27.25	2.040	1.660	1.780	2.390
12	31.87	4.690	4.190	2.520	3.790
13	34.87	4.190	6.010	2.580	2.940
14	33.18	5.180	4.620	2.910	3.370
15	17.5	3.220	2.160	2.070	2.680
16	28.28	2.850	2.840	1.990	3.320
17	31.8	4.940	4.210	3.440	4.750
18	54.56	2.680	4.890	1.620	3.390
19	27.58	4.190	3.860	2.840	4.220
20	27.92	3.890	3.720	2.380	4.490
21	19.15	1.990	3.060	2.350	3.420
22	30.17	2.850	1.370	1.220	2.940
23	26.86	3.570	2.700	2.130	1.920
24	19.32	2.420	1.660	2.750	2.860
25	29.83	2.980	3.740	1.230	2.750
26	41.89	2.650	4.180	2.270	3.320
27	28.41	4.960	4.200	3.340	3.740
28	27.71	5.160	4.060	3.740	4.170
29	34.51	4.870	4.700	1.740	3.070
30	27.93	2.630	1.570	1.690	3.160
31	27.28	2.610	2.350	3.130	3.530
32	24.2	3.820	4.370	2.360	3.990
33	44.28	2.940	2.370	2.980	3.910
34	35.12	2.770	3.500	1.070	2.880
35	46.87	2.090	2.850	1.340	2.240
36	32.49	3.570	2.600	1.510	3.260
37	30.3	4.190	3.170	2.980	3.400
38	25.68	4.340	1.860	2.470	3.090
39	35.89	3.610	1.560	1.210	3.010
40	27.65	2.020	1.870	1.070	1.570
41	29.58	4.050	4.340	1.800	3.420
42	22.23	4.570	3.720	2.840	3.540
43	34.18	2.910	2.550	2.400	3.110
44	24.96	2.550	0.500	1.050	2.620
45	28.84	3.170	2.440	3.610	3.020
46	26.91	3.680	2.580	1.080	3.400
47	49.82	4.670	5.280	2.070	2.420
48	42.49	4.870	2.370	2.020	3.250
49	29.58	2.110	2.390	1.750	2.430
50	26.17	5.390	4.310	2.420	3.610
51	28.83	3.100	1.570	1.910	2.250
52	30.35	3.820	1.970	2.540	2.690
53	27.31	2.840	2.740	1.770	2.080
54	33.09	4.370	4.350	1.680	2.460
55	23.57	5.520	4.710	2.600	3.650
56	24.66	3.670	1.610	2.880	3.140
57	40.03	4.590	3.610	2.480	3.750
58	35.97	1.860	3.230	1.120	3.000
59	34.86	2.990	2.960	2.490	3.750

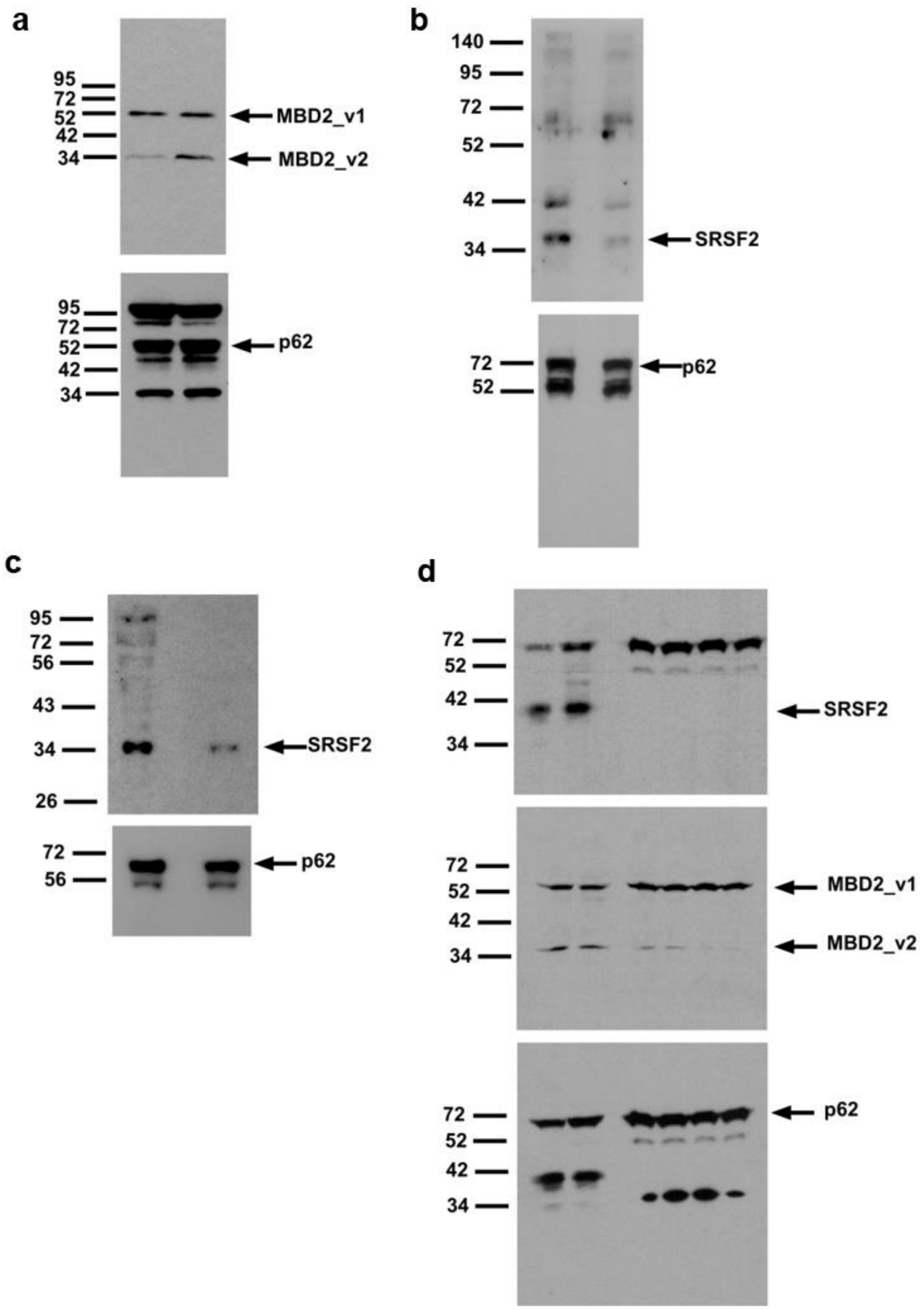

Fig. S1. Full-length scanned images of immunoblot film. Panels here correspond to cropped bands in: **(a)** Fig. 3b, **(b)** Fig. 4b top, **(c)** Fig. 4b lower, and **(d)** Fig. 4c. Protein ladders were used to estimate molecular weight in kilodaltons and are represented at the left of each panel. The antibody-targeted protein is indicated along the right side each panel.

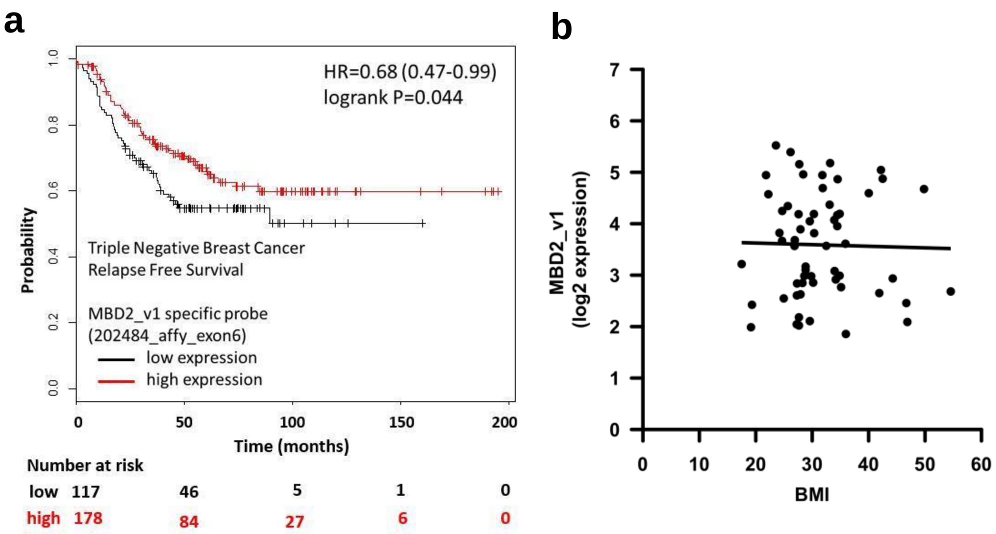

Fig. S2. Testing for associations between TNBC tumor expression of full-length MBD2 isoform MBD2_v1 **and patient outcomes and BMI. (a)** Analysis was performed with the online KM Plotter database, using a logrank test of association between relapse-free survival and MBD2_v1 transcript level. The number of subjects at risk at different time points is indicated below the x-axis. **(b)** Testing for transcript level associations with BMI, was done using a separate gene expression microarray dataset generated from TNBC specimens (n= 59) collected at the Karmanos Cancer Institute, Detroit, MI, where BMI data corresponding to deidentified samples was available. The association between BMI and MBD2_v1 expression was tested using linear regression analysis (*P* > 0.05, not significant).

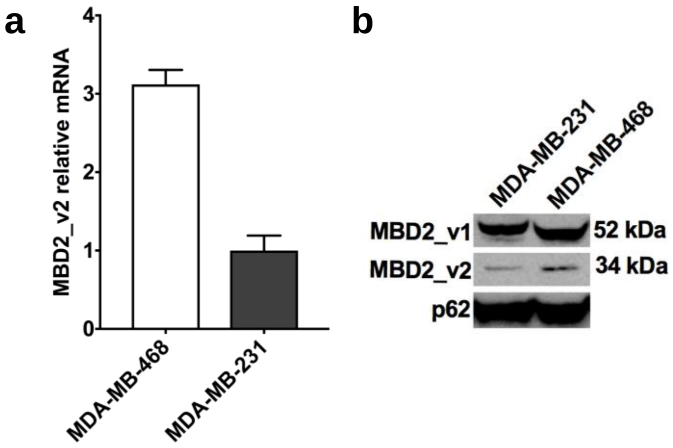

Fig. S3. MBD2_v2 expression in TNBC cell line cultures prior to mouse inoculation. (a) Comparison of MBD2_v2 expression levels in MDA-MB-468 and MDA-MB-231 cell cultures by semiquantitative RT-PCR, and **(b)** immunoblot analysis.

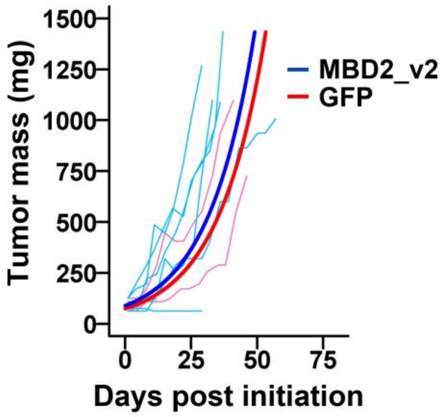

Fig. S4. MBD2_v2 overexpressing and GFP expressing tumor growth curves. Growth curves for tumors formed by MBD2_v2 overexpressing and GFP expressing control MDA-MB-231 cells in mice on the control formula diet. Tumor mass was plotted for each tumor over the course of the 150 day experiment and modeled growth curves (bold) are superimposed. A generalized least squares test was used to calculate a *P* value (*P* > 0.05).

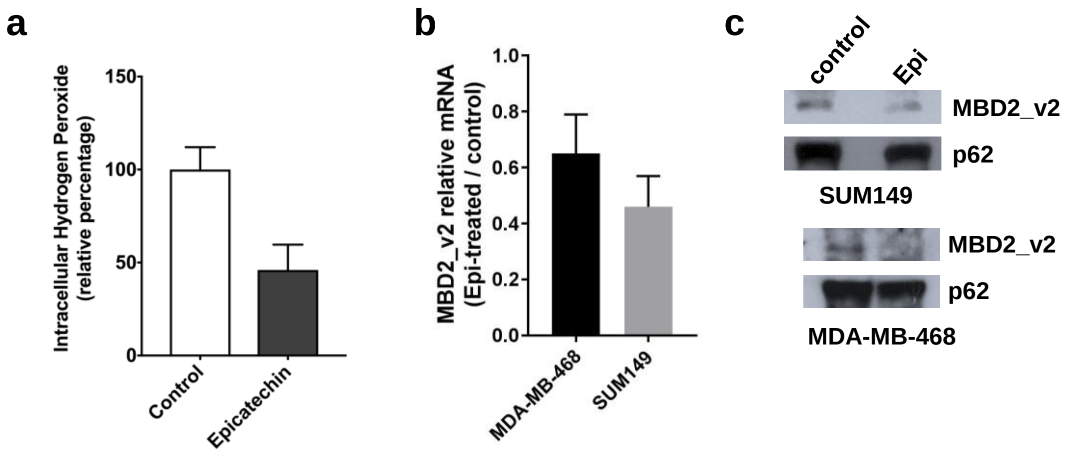

Fig. S5. Effect of (–)-epicatechin antioxidant treatment on ROS and MBD2_v2 levels in TNBC cell cultures. (a) The effectiveness of the (–)-epicatechin preparation to decrease hydrogen peroxide levels was confirmed using MDA-MB-468 cells, 48 hour 120µM treatment, and the MAK164 Intracellular hydrogen peroxide assay (Sigma-Aldrich). Results are the mean of 3 independent experiments ± s.e.m. **(b)** The effect of (–)-epicatechin (Epi) antioxidant treatment (48 hours, 120µM) to reduce readily MBD2_v2 levels in MDA-MB-468 and SUM149 TNBC cell lines, measured by semiquantitative RT-PCR analysis of RNA; **(c)** and by immunoblot analysis of protein lysates. Semiquantitative RT-PCR (mean fold-change for sets of 3 technical replicates ± s.d.) and immunoblot data are representative of 2 independent experiments for a each cell line.

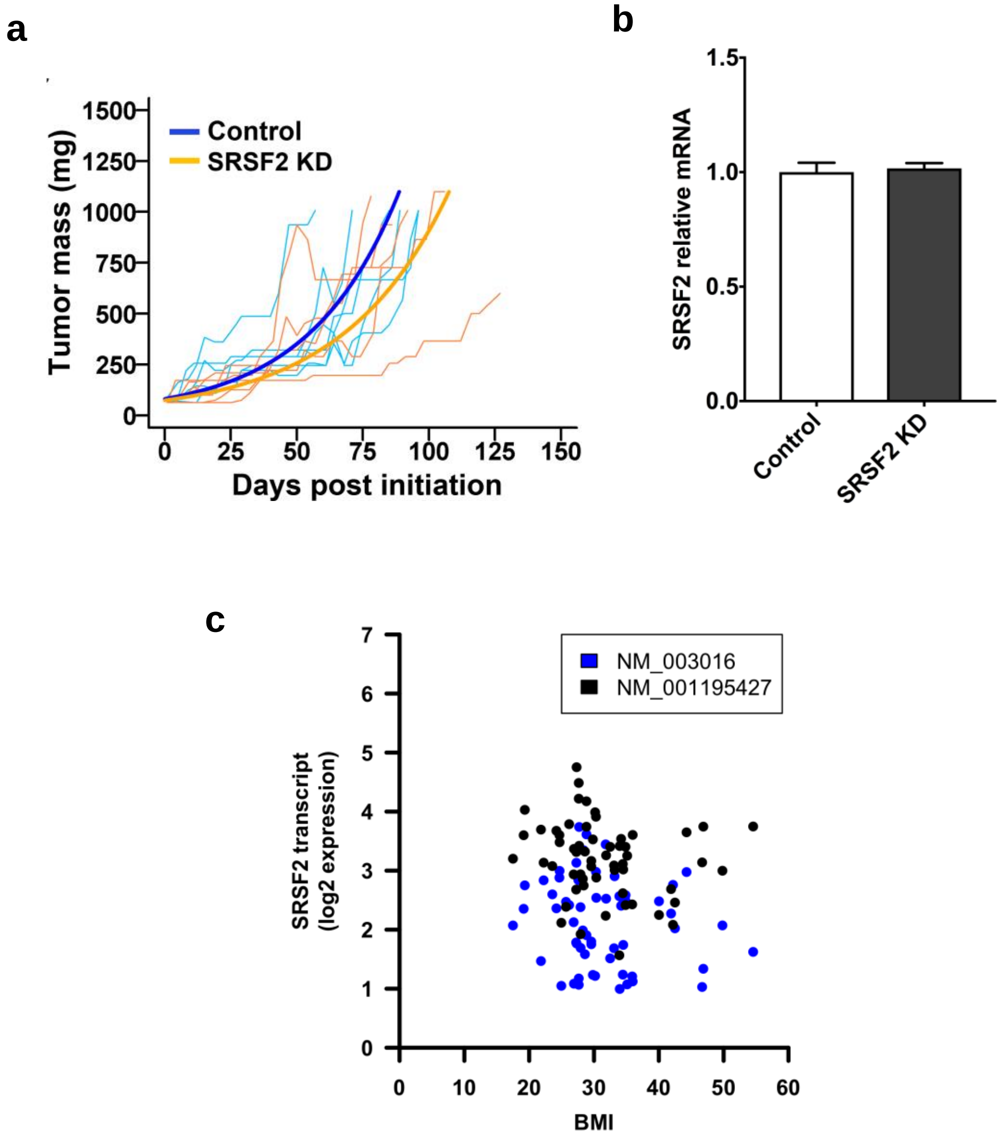

Fig. S6. SRSF2 knockdown tumor levels and growth curves, and patient tumor SRSF2 expression related to BMI. (a) SRSF2 knockdown and nonsilencing MDA-MB-468 cell line-derived tumor growth curves. Tumor mass was plotted for each tumor over the course of the 150 day experiment and modeled growth curves (bold) are superimposed. A generalized least squares test was used to calculate a *P* value ($P > 0.05$). **(b)** SRSF2 levels in tumors formed by SRSF2 knockdown and nonsilencing vector control MDA-MB-468 cells harvested from DIO mice (assessed by semiquantitative RT-PCR analysis). **(c)** Graph of patient tumor SRSF2 transcript expression and relationship with BMI (KCI dataset). 2 of 2 translated variants (NCBI Refseq IDs) are plotted. There is no significant association between the variables.

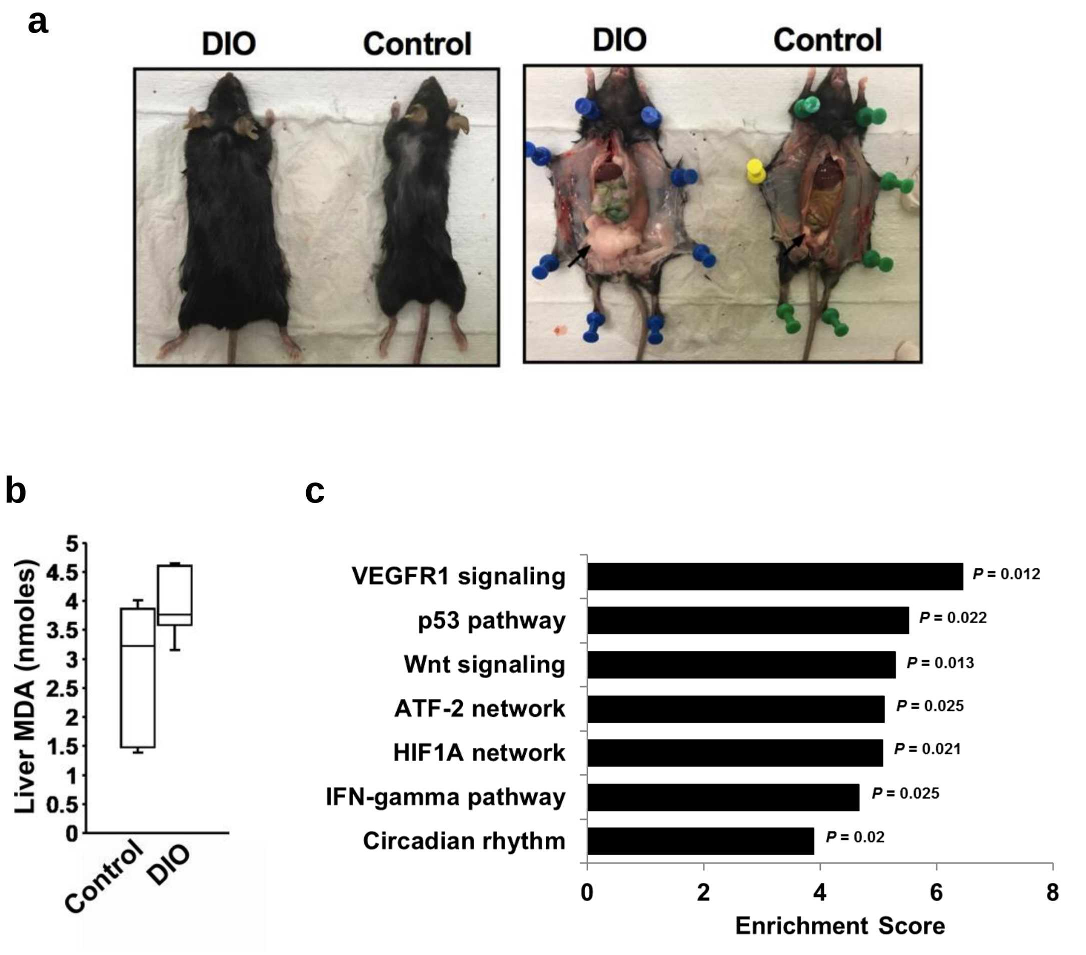

Fig. S7. Visceral adiposity, oxidative stress levels and enrichment of signaling pathway genes in tumors comparing DIO and control mice. (a) Representative DIO specimen exhibiting increased visceral adiposity relative to lean control mouse. These examples were humanly euthanized when tumor burden end-point was reached 100 days post inoculation. **(b)** Liver malondialdehyde (MDA) levels (an indicator of systemic oxidative stress) in DIO and control mice (6 randomly selected per group). **(c)** Tumor signaling pathways impacted by DIO. Genome-wide expression analysis was performed to compare MDA-MB-468 tumors harvested from DIO mice (n=3, randomly selected) with those harvested from lean control mice (n=3). The Enrichr tool and NCI-Nature Pathways library were applied to the significant differentially expressed gene set ($P < 0.01$) to identify significantly over-represented signaling pathways. The P value of overlap and top ranking Enrichment Scores, a significance value optimized for and calculated by the Enrichr tool, are reported.

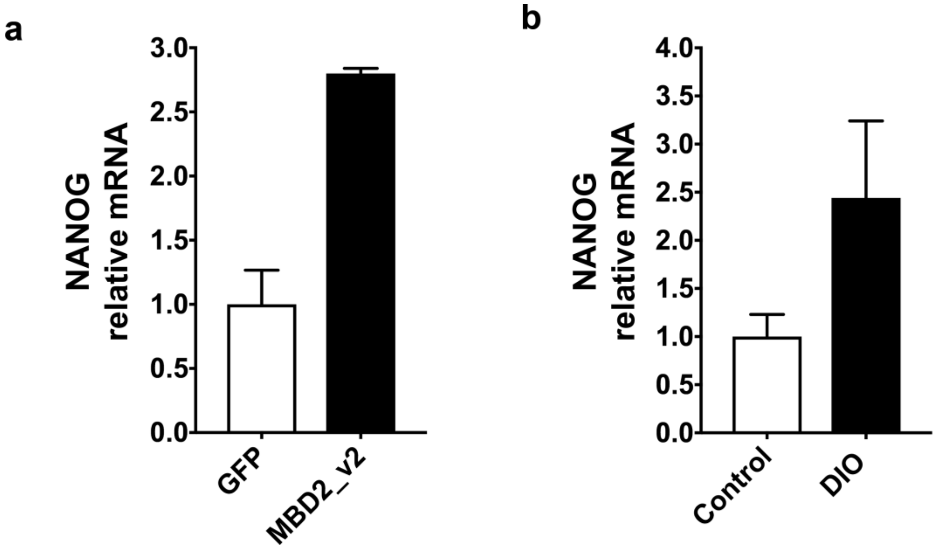

Fig. S8. NANOG in TNBC cell line cultures and tumors. (**a**) NANOG gene expression in cultures of MBD2_v2 overexpressing MDA-MB-468 cells compared to GFP-expressing controls by semiquantitative RT-PCR analysis ($P < 0.001$, Welch's t-test). *Bars*, ± s.d. for 3 technical replicates. (**b**) Comparison of NANOG expression in MDA-MB-468 tumors harvested from DIO (n=3) and control (n=3) mice by semiquantitative RT-PCR ($P < 0.01$, Welch's t-test). *Bars*, ± s.e.m. NANOG was similarly observed to be upregulated in tumors from DIO mice by microarray data analysis ($P \leq 0.05$, accessible at GSE114604).
